# A mixed‐methods characterisation of patient safety incidents by primary eye care practitioners

**DOI:** 10.1111/opo.13030

**Published:** 2022-07-31

**Authors:** Elinor MacFarlane, Andrew Carson‐Stevens, Rachel North, Barbara Ryan, Jennifer Acton

**Affiliations:** ^1^ School of Optometry and Vision Sciences Cardiff University Cardiff UK; ^2^ PRIME Centre Wales, Division of Population Medicine, School of Medicine Cardiff University Cardiff UK

**Keywords:** eye, eyecare, incident, optometry, patient safety, primary care, public health

## Abstract

**Purpose:**

Patient safety in eye health care is an underdeveloped field of research. A patient safety incident occurs when an unintended incident happens that could have (or did) lead to harm. To enable learning from patient safety incidents in optometry, a characterisation of commonly experienced safety incidents is needed to identify options to improve the quality of care. This study aimed to characterise eye health‐related patient safety incidents from the perspective of eye care practitioners.

**Methods:**

At a national conference in Wales, 56 eye care practitioners participated in a stakeholder workshop on eye care‐related patient safety incidents. Participants were asked to suggest patient safety incidents that have occurred, or based on their experience, could occur in optometric practice. Using the nominal group technique, participants voted on the incident they perceived could cause the most harm and the incident observed most frequently in practice. Framework analysis supported identification of themes about the nature and outcomes of incidents in eye care.

**Results:**

Diagnostic incidents were perceived to be the most severe (highest number of ‘severity votes’, *n* = 38), whilst administration‐related incidents were most frequent (highest number of ‘frequency votes’ *n* = 39). Four themes were identified which are as follows: inappropriate clinical decision‐making; delayed or missed referral of patients to general medical practitioners or ophthalmologists; compromised communication with other practitioners or patients and delays in receiving eye care. The results suggest that incidents relating to inappropriate clinical decision‐making could result in the most severe harm to patients but may not occur frequently.

**Conclusions:**

Diagnostic‐ and administrative‐related incidents pose clear challenges for improvement in quality and safety of care. The breadth of themes reflecting the nature and outcomes from unsafe eye care highlights the complexity underpinning incidents and the burden to patients. This work has informed the content of an all‐Wales incident report form for primary eye care practitioners.

**Key points**

- We present the first characterisation of perceived patient safety risks based on the experiences of eye care practitioners, which is important given the increased scope of practice in primary care optometry.
- In collaboration with policymakers, the findings contributed to the development of a new patient safety incident reporting system for staff working in primary eye care.
- Incident reporting that can inform actions to improve patient outcomes is fundamental to growing and sustaining the culture of safety as part of clinical governance in eye care.

## INTRODUCTION

Patient safety is a discipline that has developed to address healthcare‐related (iatrogenic) harm and improve the quality of care for patients.^1^ At the Fifty‐fifth World Health Assembly in 2002, the World Health Organisation (WHO) identified unsafe health care as an avoidable problem, and urged member states to establish and strengthen science‐based systems for the reporting of incidents to enhance their healthcare system and improve safety.^2^ Two decades later, the Seventy‐Fourth World Health Assembly adopted a 10‐year action plan outlining how healthcare systems can and should do more to protect patients.^3^

Patient safety research and culture is well developed in general health care, and extensive research has been undertaken in secondary care settings. One method used for researching the safety of care being delivered involves evaluating the experiences of healthcare professionals and staff through reports about patient safety incidents that have occurred in a service. A patient safety incident is defined by the UK National Health Service (NHS) as ‘any unintended or unexpected incident that could have or did lead to harm for one or more patients receiving healthcare’.^4^ By understanding how patients did or could have come to harm, improvements can be made to prevent changes from reoccurring. Setting priorities for targeted safety improvements has been essential in improving the quality of care that is being delivered.

To our knowledge, there have been no studies of eye health‐related safety in primary care settings, and few studies in tertiary^5^ or secondary‐level^6^, ^7^, ^8^, ^9^, ^10^, ^11^, ^12^ services including incidents relating to eye surgery,^5^, ^6^, ^7^, ^8^, ^9^ patients being lost to follow‐up,^10^ anti‐VEGF (vascular endothelial growth factor) medication^11^ and nurse treatment of eye emergencies.^12^ Common patient safety incidents that were reported across studies included delays,^8^, ^10^, ^11^ wrong eye/patient,^7^, ^8^, ^11^ wrong intraocular lens implant^5^, ^6^, ^8^ and wrong medication.^7^, ^11^

In 2014, the sight loss of half of the 1.8 million people in the United Kingdom living with vision impairment was deemed to have been ‘avoidable’.^13^ In addition, there is compounding evidence that delays to receiving care and/or treatment of eye conditions leads to irreversible sight loss for patients in the United Kingdom.^14^ The Welsh government publishes monthly reports on the waiting times for patients assessed to be at the greatest risk of irreversible harm or a significant adverse outcome if their target date (typically 26 weeks)^15^ was missed. In January 2020, one‐third of the highest health risk factor (R1) patients were more than 25% past their target dates,^16^ and by January 2021, this figure had risen to 57%.^17^ These data reinforce the importance of a robust incident reporting system to learn from the harm that is arising from delays, along with any other incidents.

The rationale for the study was consistent with the increased scope of clinical practice in the United Kingdom, in which a restructuring of primary eye care services has led to the delivery of enhanced optometric services in the community. The study also aligned with early planning discussions for an incident reporting system for use in primary eye care in Wales to enable learning from incidents occurring in optometric practice, to inform system development. This study aimed to characterise patient safety incidents occurring in primary eye care by identifying incidents that occur in optometric practice. We sought to establish initial priorities to address the most frequent and harmful patient safety incidents from the perspective of primary eye care practitioners working in Wales.

## METHODS

### Study design

This study used a convergent mixed‐methods design,^18^ which involved the collection of both quantitative and qualitative data, and analysis and integration through the merging of results to compare the findings. This research is informed by an interpretivist/constructivist approach, which is based on the ontology that reality is socially constructed.^19^ The approach attempts to obtain multiple perspectives, from stakeholders with a range of experiences, in order to make sense of participants' reality of the phenomena being studied. In this study, the approach involved seeking the participants' experiences of incidents in practice to understand patient safety in eye care from those involved in the delivery of care.

### Setting and sample

Optometrists, dispensing opticians, Welsh Optometric Committee members and government representatives attended the ‘A Healthier Wales: Transformation of Eyecare Services’ conference in February 2020. Data were collected as part of a stakeholder workshop that aimed to encourage participants to recognise patient safety incidents that need to be reported.

#### Workshop: reducing harm and improving quality of care

The workshop was designed using the UK Royal College of General Practitioners (RCGP) Reporting and Learning Guide.^20^ This is based on the work of Carson‐Stevens et al.,^21^ to reinforce the importance of reporting safety incidents as it is integral to learning and improving the quality of care in primary care settings. As there was no existing system for primary care‐based optometrists to report safety incidents in Wales at the time of data collection, the information delivered in the stakeholder workshop emphasised the wider purpose of incident reporting in healthcare to improve the quality of care. A new (first draft) incident report form for use in primary eye care in Wales was debuted at the workshop and iteratively developed based on the proceedings.

### Data collection

The definition of a patient safety incident was explained to the workshop participants, then in small groups of up to seven individuals, participants were given the opportunity (by author BR, facilitator through their role as Chair of the Welsh Optometric Committee) to suggest safety incidents that they had seen occur or could occur in optometric practice. Suggested incidents were discussed and scribed on to Post‐it ***®*** Notes before the groups allocated them to one of five pre‐defined incident categories (adapted from RCGP, 2017) based on a large mixed‐methods study of safety incidents in general practice: medication, diagnosis and clinical investigation, communication, equipment or administration. Participants were instructed to categorise communication incidents that could occur within practice under ‘communication’, and communication incidents that could occur out of practice or across healthcare services under ‘administration’.

**FIGURE 1:**
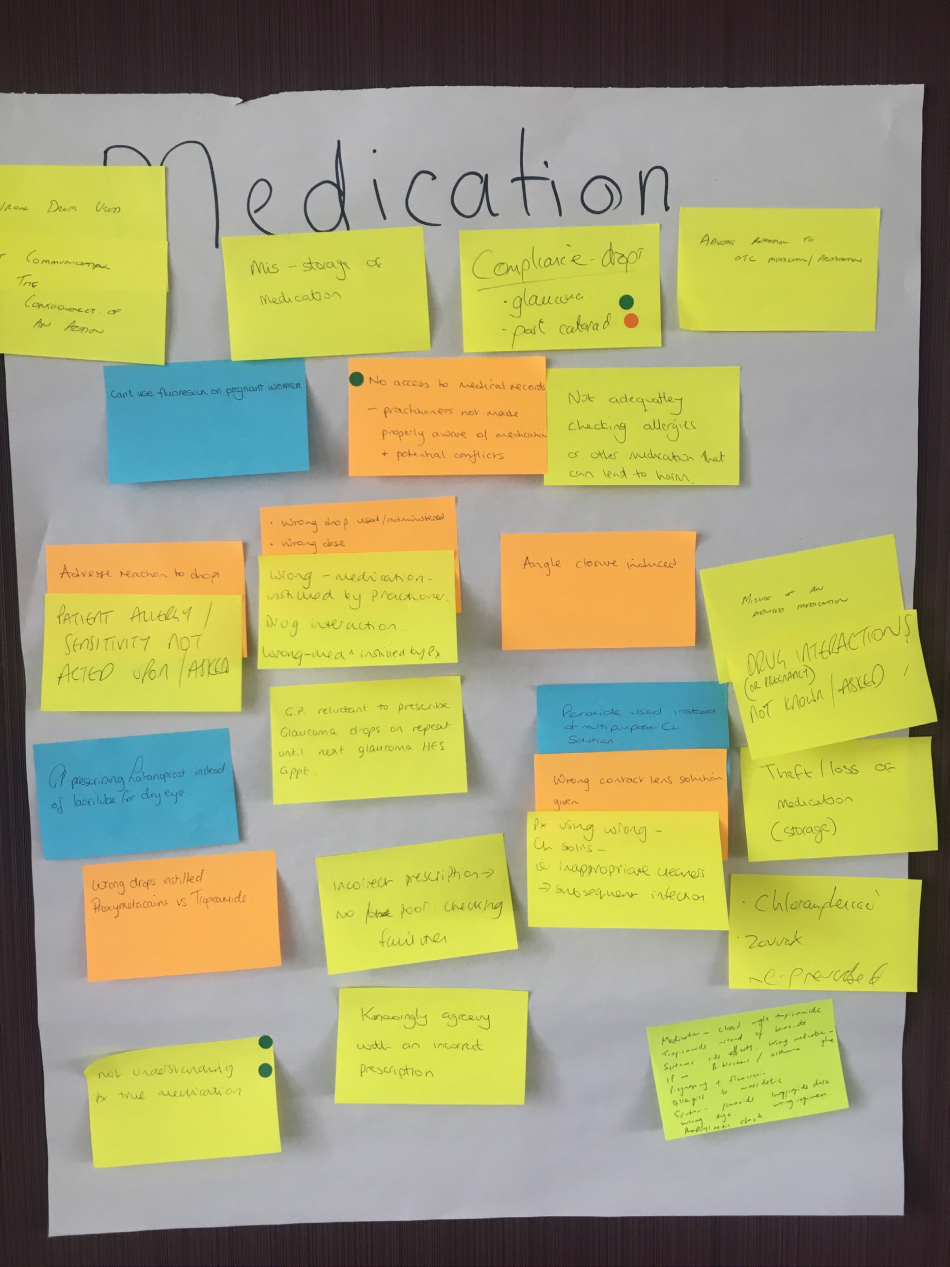
Suggested incidents and participant votes in the ‘medication’ category.

**FIGURE 2:**
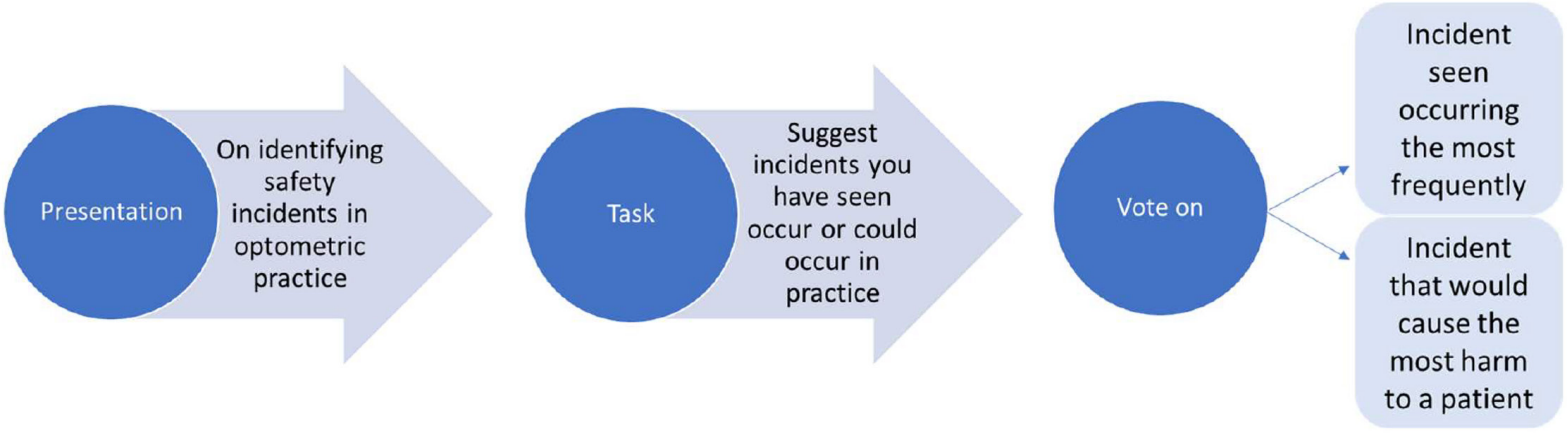
Data collection process.

Following the categorisation task, the suggested incidents from each group were collated by category, and participants were given the opportunity to read the suggested incidents from other groups. After this, participants took part in a nominal group technique process and were asked (by author EM, facilitator and researcher) to identify the most frequently occurring incidents and the incidents that could cause the most severe harm to patients.^22^ The nominal group technique is a straightforward method to measure consensus between a large group of participants. Each participant placed a vote against the incident that they perceived occurs most frequently and would lead to the most severe outcomes. Voting was undertaken using two coloured stickers, one denoting the incident that the participant had observed to occur most frequently in practice, and the other denoting the incident that had the greatest potential to cause harm to a patient (Figure 1). Photographs were taken following the workshop to record the data. The structure of the workshop can be seen in Figure 2.

### Data analysis

#### Demographic analysis

Demographic data for the eye care practitioners (sex, number of years since professional registration and speciality qualifications) were obtained from the conference provider following ethical approval. Existing data on eye care practitioners in Wales were obtained (personal communication, Dr Nik Sheen, Health Education and Improvement Wales, 14 January 2021) and presented alongside the demographic data of the eye care practitioner workshop participants, to demonstrate workforce representativeness. The age of the eye care practitioner participants was inferred from their number of years since registration and the expected professional qualifying age for a population of optometrists, calculated from the median age of students starting degrees in optometry (aggregated student intake data 2017–2020, School of Optometry and Vision Sciences, Cardiff University). This was then compared with the age distribution of the population of eye care practitioners in Wales.

#### Quantitative analysis

Descriptive statistics were generated for both the demographic data and the participant votes on the most frequent and severe suggested incidents: hereinafter referred to as ‘frequency’ and ‘severity’ votes.

#### Qualitative analysis

The qualitative data (text on Post‐It notes) were uploaded within the incident categories for analysis to NVivo data management software (version 12, QSR International, qsrinternational.com). Framework analysis^23^ was used to address contextual questions, which in this case involved identifying the nature of the participants' experiences involving incidents, and to generate, inductively, hypotheses about the nature of patient safety incidents in eye care in Wales. A thematic framework was identified following familiarisation of the data, and the incidents were coded (indexed) before being charted to support interpretation of findings.

#### Locus of control

Each of the incidents were additionally categorised (by author EM) according to whether the incident was deemed to have been ‘avoidable’,^24^ and if so, where the perceived ‘control’ of the incident lay (within a practitioner's own control or either another practitioner or the patient's control), or if not, they were deemed to be ‘out of control’. Some incidents were placed in more than one category.

#### Severity of harm

Two registered optometrists with academic backgrounds (authors JA and RN) classified the potential level of harm that could result from the submitted incidents. Discussions were held to determine the levels of disagreement, with a third optometrist available in the event that consensus was not reached. If an incident had the potential to cause a range of harm, it was counted in each of the levels included in the given range. This ensured that the proportion of harm in each category was representative of the potential harm severity of the incidents overall.

#### Mixed‐methods integration

The quantitative data (severity votes) were integrated with the qualitative data (themes from description of incidents and harm severity of incidents),^18^ to compare findings and identify the outcome of the integration. This could include the following: *confirmation* (in which the findings from the different data sources confirm the findings of the other); *expansion* (in which the findings from the different data sources have both overlapping and non‐overlapping themes that expand upon the previous knowledge) or *discordance* (in which the findings from the different data sources contradict the findings of the other).^25^

### Ethical considerations

Ethical approval was sought retrospectively and approved by the School of Optometry and Vision Sciences Research and Audit Ethics Committee at Cardiff University (project number: 1546) on the basis of analysing anonymised (non‐identifiable) data generated during the workshop and that transferable learning was likely from secondary analysis of these data.

## RESULTS

### Demographic data

Data were collected from 56 participants: 45 were optometrists, seven were dispensing opticians and four were health board representatives or managers. Demographic data were obtained from the eye care practitioner participants. The sex distribution of the optometric workforce in Wales is 52% women and 48% men, while the distribution of eye care practitioner workshop participants was 63.5% women and 36.5% men. The sample therefore contained a higher proportion of women (11.5% more) than the optometric workforce population in Wales. The age distribution of the Welsh optometrist workforce was consistent with the estimated age of eye care practitioner workshop participants, as shown in Figure S1, with slightly more (5%) participants in the 41–50 years age range and less (0%) in the 71 and over age range. The percentage of eye care practitioner participants holding additional qualifications was 21%, compared with 9% of eye care practitioners in Wales. The distribution of the higher qualifications held by the eye care practitioner participants was similar to that of the optometric workforce in Wales (Figure S2).

### Frequency and severity of incidents

**FIGURE 3:**
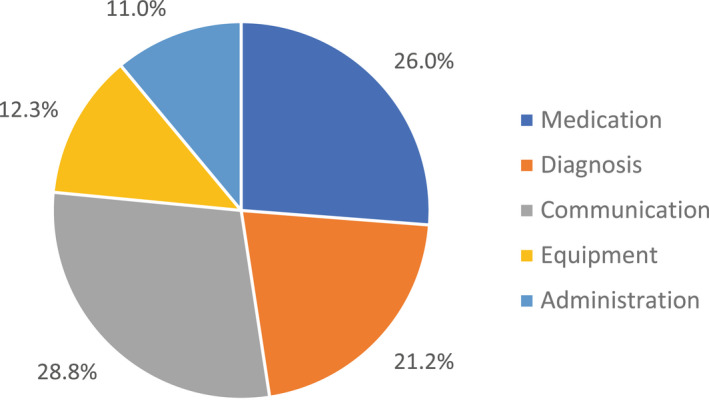
Percentage of suggested incidents within each of the five categories.

**FIGURE 4:**
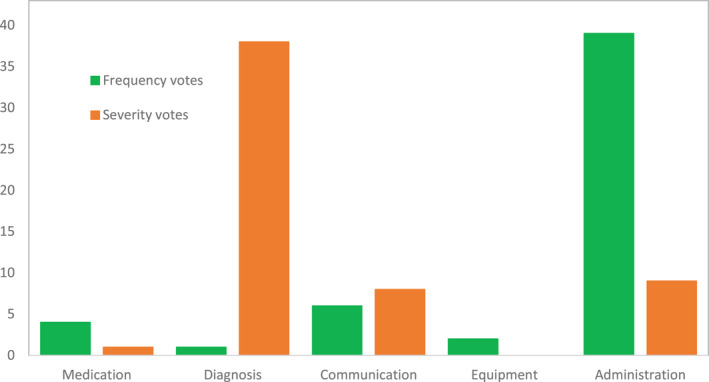
Number of ‘frequency’ and ‘severity’ votes for incidents in each of the five categories.

A total of 146 incidents were suggested overall. The number of suggested incidents and the number of votes assigned to the incidents within each category are summarised in Figures 3 and 4, respectively. Specific incidents within each category are listed in Table S1.

The categories with the most and fewest incidents suggested were communication (*n* = 43) and administration (*n* = 16), respectively (Figure 3). Incidents in the administration category received the highest number of ‘frequency’ votes (*n* = 39), meaning that participants saw these incidents occurring most frequently in practice. Incidents in the diagnosis and clinical investigation category received the highest number of ‘severity’ votes (*n* = 38), indicating that participants perceived these incidents to have the highest potential to cause harm to patients.

Seventy‐five per cent (*n* = 39) of all ‘frequency’ votes were allocated to incidents in the administration category, and 68% (*n* = 38) of ‘severity’ votes were allocated to incidents in the diagnosis and clinical investigation category. The data show that participants considered administration incidents to be the most frequently occurring in optometric practice in Wales, and within this category, ‘lost to follow up’ incidents received 38 ‘frequency’ votes. The ‘lost to follow up’ incidents in the administration category also received nine ‘severity’ votes, the second highest number of votes for an incident category. Participants considered incidents in the diagnosis and clinical investigation category to have the greatest potential to cause severe harm to patients, as incidents due to ‘missed pathology’ in this category received 22 votes and ‘unable to access eye casualty when required’ received seven votes.

### Themes from descriptions of incidents

Four themes were identified from the suggested incidents: ‘inappropriate clinical decision making’, ‘delayed or missed referral of patients to GPs or ophthalmologists’, ‘communication with other practitioners or patients’ and ‘delays in receiving eye care’. In addition, a sub‐theme, ‘patient adherence to guidance’ was generated within the ‘communication with other practitioners or patients’ theme. Three of the themes were related to the nature of incidents: inappropriate clinical decision‐making (theme 1), delayed or missed referrals (theme 2) and issues arising from communication problems (theme 3). A further theme related to the outcome of delayed patient care (theme 4). A summary of the qualitative data and a graph displaying the allocation of incidents in each category to the themes are located within the supplementary information (Table S2, Figure S3).

#### Themes relating to the nature of incidents

##### Theme 1: Inappropriate clinical decision‐making

The ‘inappropriate clinical decision making’ theme described incidents in which a situation had arisen from an error in a practitioner's clinical judgement. Most of these incidents were related to problems involving medication (*n* = 29/80). Examples of incidents included the following: not asking about or acting upon patient allergies, not dilating a patient when clinically necessary, practitioners not keeping up to date with new techniques and their interpretation (e.g., optical coherence tomography [OCT] imaging) and patients not being triaged at the point of referral or not being triaged sufficiently quickly.

##### Theme 2: Delayed or missed referral of patients to GPs or ophthalmologists

Incidents were placed under the ‘delayed or missed referral of patients’ theme if they related to problems with either incoming or outgoing referrals. Many of these incidents were underpinned with challenges in communication (*n* = 14/35). Examples of incidents included practitioners not referring a patient, not having access to medical records (resulting in lack of awareness of medication history or potential interactions), sending referrals to the wrong GP surgery, having issues when faxing referrals and breaching the recommended timescale for referrals.

##### Theme 3: Compromised communication with other practitioners or patients

Multiple incidents were related to communication problems between practitioners or with a patient (*n* = 26/75). Examples of incidents included practitioners not communicating the consequences of an action, patients who have not returned for a review, patients who have not been followed up by the practice, failure to make arrangements to repeat tests when instrumentation was not in working order and receptionists issuing prescriptions that had not been checked or signed, leading to incorrect spectacles being manufactured by an external provider.

### Sub‐theme: patient adherence to guidance

The ‘patient adherence to guidance’ sub‐theme included three incidents that related to medication problems, such as patients following the wrong regimen for medication or using the wrong contact lens solution. Some were concerned about patient memory or their mental capacity. Nine incidents related to communication problems affecting patient adherence to guidance on their specified regime for drop instillation, use of contact lens solution or patient identification of new symptoms that would require a consultation.

#### Theme relating to an outcome of incidents

##### Theme 4: Delays in receiving eye care

Incidents were placed under the theme, ‘delays in receiving eye care’, if they described a situation resulting in a delay to a patient's care, potentially leading to (further) harm. Most incidents resulted from errors in diagnosis and clinical investigation (*n* = 23/75). Examples of such incidents included ophthalmic medications being instilled into the wrong eye, not making referrals when clinically indicated, administrative issues resulting in a referral letter not being sent or the patient being lost to follow‐up and poorly calibrated equipment resulting in the requirement for repeated appointment/visits.

### Locus of control

Whether an incident was perceived to have been avoidable or not informed the charting of the incident's ‘locus of control’, including three categories: own control, other's control and/or out of control (unavoidable).

**FIGURE 5:**
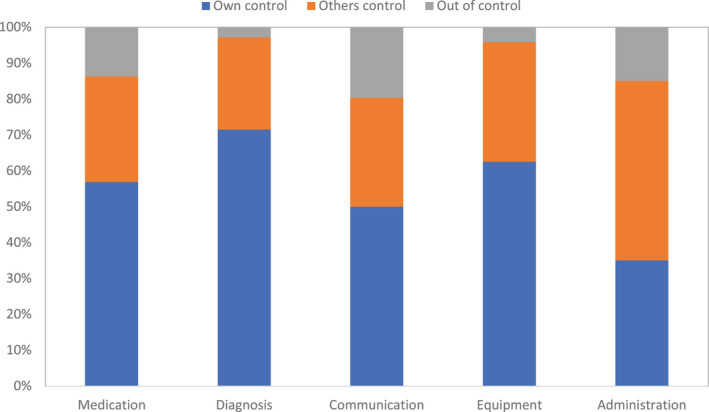
Number of incidents, according to the locus of control, represented as the percentage of incidents within each incident category.

Figure 5 shows the proportion of incidents allocated according to locus of control in each incident category. Incidents deemed ‘out of control’ varied across the incident categories, for example, “mental capacity issues” in the communication category. Many incidents were deemed to be ‘within control’, either under ‘own control’ (e.g., failure to look at test results) or ‘other's control’ (e.g., a GP changing drug requests). The administration category contained the lowest number (*n* = 7) and proportion of incidents that were charted under ‘own control’. A summary of specific incidents allocated by locus of control is provided in Table S3.

### Harm severity

**FIGURE 6:**
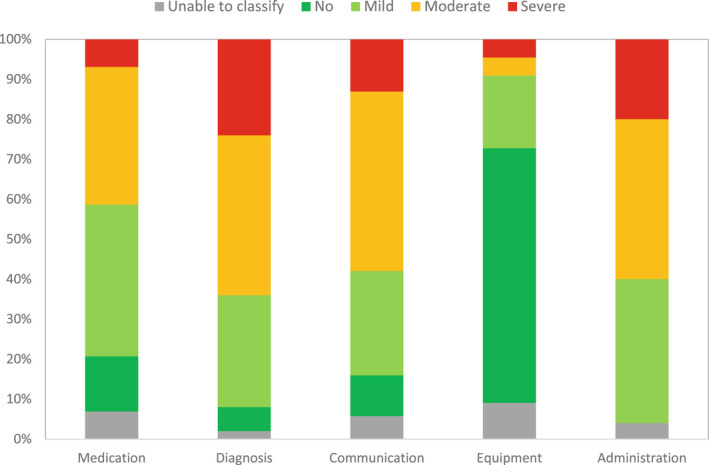
Number of incidents, at each level of harm severity, represented as a percentage of the total incidents classified within each incident category.

There were no resulting disagreements between the two initial raters following the discussions on the classification of harm severity for the suggested incidents. Classification was subject to clinical opinion and judgement and referred to a potential range of scenarios for each suggested incident. Those incidents identified as spanning a range of harm severity levels were counted as a separate incident in each of the harm levels allocated in Figure 6. A summary of incidents allocated within each harm classification is provided in Table S3.

Figure 6 shows the proportion of harm charted for the submitted incidents, to highlight the potential severity of incidents allocated within each incident category. The categories with the largest and smallest proportion of incidents that had the potential to cause severe harm were ‘Diagnosis and clinical investigation’ (24%) and ‘Equipment’ (5%). The ‘Equipment’ category also had the largest proportion of ‘no harm’ incidents suggested (63%). The categories with the smallest proportion of incidents that would cause no harm were ‘Administration’ (0%) and ‘Diagnosis and clinical investigation’ (6%).

### Mixed‐methods integration

**FIGURE 7:**
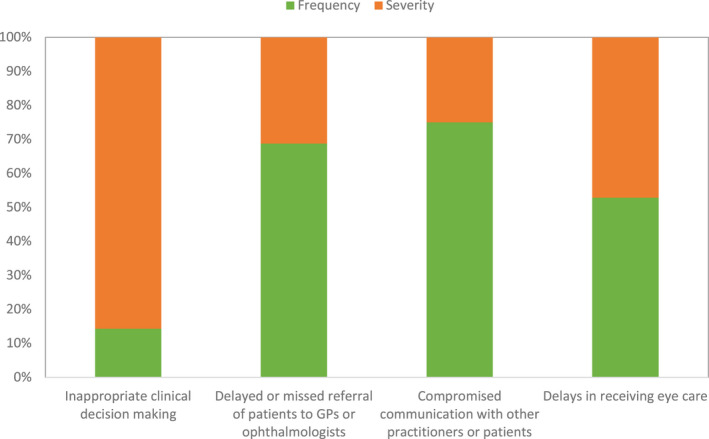
Proportion of frequency and severity votes for incidents within each theme.

Figure 7 shows the proportion of ‘frequency’ and ‘severity’ votes for incidents within each of the four themes from the description of incidents. Participants ranked incidents relating to inappropriate clinical decision‐making as having the highest proportion of severe outcomes (*n* = 36, 88%) relative to those occurring frequently in practice (*n* = 5, 12%). Incidents relating to communication with other practitioners or patients had the highest proportion occurring frequently in practice (*n* = 45, 75%) and the lowest proportion of severe outcomes (*n* = 15, 25%). The integration analysis demonstrates expansion, as the results suggest that incidents relating to inappropriate clinical decision‐making could result in the most severe harm to patients, but may not occur frequently in practice. In addition, incidents relating to communication with other practitioners or patients could occur most frequently in practice, but are less likely to result in severe harm to patients. Incidents relating to delays in receiving eye care had the overall largest numbers of votes (47 ‘frequency’ votes and 42 ‘severity’ votes) and were perceived to have the most equal proportion of occurring frequently in practice (53%) and severe outcomes (47%). The integration analysis further demonstrates expansion, given the additional information that incidents resulting in delays were voted equally to be the most severe and frequently occurring.

## DISCUSSION

The findings indicate that the majority of workshop participants reported administration‐related incidents to occur most frequently in practice and such incidents were the second most severe. Diagnosis‐related incidents were perceived to cause the most severe harm to patients. Four themes were identified as follows: inappropriate clinical decision‐making, delayed or missed referral of patients to GPs or ophthalmologists, compromised communication with other practitioners or patients and delays receiving eye care. One sub‐theme was identified within the compromised communication theme which related to patient adherence to guidance. Importantly, the suggested incidents reported were often within the control of primary eye care practitioners, signalling the need for improvement of safety in eye health at a local level. It was found that incidents resulting in delayed care were perceived to occur both frequently and severely, which suggests that delays have a high potential to cause harm to many patients.

Incident reporting systems can help establish priorities and focus resources to investigate incidents further. The field of human factors recognises the interactions between human, social, organisational and technical factors in achieving workplace goals and the related impact on patient safety,^26^ and is included in the WHO Global Patient Safety Action Plan for 2021–2030.^27^ A structured approach to understanding human factors^28^ should be used to maximise their identification, and to understand relationships between factors and how they can contribute to similar incidents in the future. Practitioners can use frameworks such as the Systems Engineering Initiative for Patient Safety (SEIPS 2.0)^29^ to help identify holistically and comprehensively the likely range of factors contributing to incidents. As healthcare systems are inherently complex, the analysis of incident reports alone is limited, and additional methods are required to understand the human factors fully. In doing so, practitioners should not oversimplify the complex relationships between antecedents (contributory and mitigatory). From the reports, at the very least, hypotheses can be identified, which can be corroborated or amended as a greater understanding of the safety phenomenon is achieved by more in‐depth, follow‐on inquiry. For example, investigations or analysis of complimentary data sources. Sharing findings from incident report analyses can also help structure subsequent quality improvement planning discussions. Acknowledging the reports and their analyses can only provide one (and sometimes a limited) window to the system. The tacit wisdom and lived experiences of the project team can often helpfully add context and contribute nuance to plans for solutions to mitigate future similar incidents.^30^ As such, recommendations made from this study are offered as starting points on areas to begin a wider and more in‐depth enquiry.

Incident reporting can drive and make important contributions to learning that leads to change and improvement, which must be the prime overall rationale for asking staff to take the time to complete reports.^31^ Therefore, it is critical that healthcare systems develop learning cultures where staff (and even patients and members of the public) can appreciate how their contributions are enabling learning, and how it is being used to improve practice and prevent future such incidents.^32^

Incidents resulting from a patient being ‘lost to follow up’ were deemed to occur most frequently in practice and ranked the second highest for having the potential to cause severe harm to patients. This finding supports the evidence that delays are occurring in eye care,^14^, ^16^, ^17^ and that these events are causing harm to patients. Incidents involving the referral of patients were suggested under every incident category, indicating that this may be a widespread problem.

The findings highlighted diagnosis‐related incidents as the most severe. These results are consistent with those of patient safety incident studies across general health care^21^, ^33^, ^34^ that incidents arising from diagnostic errors have the largest potential to cause harm to patients. Furthermore, misdiagnosis was identified as an area of risk by the UK professional regulatory body for optical professions, the General Optical Council (GOC).^35^ In this study, incidents specifically due to ‘missed pathology’ (diagnosis‐related) were felt to be the most severe, but were not deemed to occur frequently. This finding reflects an overall trend as diagnostic incidents received the fewest number of frequency votes from the practitioners, suggesting that such incidents may not be seen very often in practice.

Incidents such as misdiagnosis were present across incident categories and can arise due to inappropriate clinical decision‐making. Inexperience can only be a plausible explanation after a multitude of factors have been considered such as working conditions, training and appraisal mechanisms and even patient factors (poor historian, downplaying symptoms, the signs or symptoms are rare for the condition). In a blame‐free learning healthcare system, those reviewing such reports and/or leading investigations to understand why a patient experienced the outcome must take into consideration the probable diverse influences on practitioners and their performance.^28^ Incidents relating to ‘compromised communication with other practitioners or patients’ were highest in the ‘Communication’ category, but were also present within the other incident categories. As communication is a key component of any healthcare system, incidents such as ‘not communicating the consequences of an action’ or ‘incomplete notes on referrals’ could be explored further to gain a greater understanding of how to optimise system performance and prevent re‐occurrence in similar contexts. Incidents that could result in delays to planned care were identified across all of the incident categories, for example a patient receiving ‘inappropriate treatment’ or not receiving a follow‐up letter. These incidents provide insight into the range of areas in which delays in care are being experienced. The identification of underlying factors can contribute to the development of targeted interventions for improvement to mitigate the risks in the system, leading to the prevention of further incidents taking place.

Our intention to identify the locus of control for each incident was to clarify the likely real‐world starting points from which to begin a wider and more in‐depth enquiry. It was noteworthy that several incidents that were charted under ‘own control’ related to current guidelines for the profession. Incidents such as ‘Not adequately checking allergies or other medications that can lead to harm’ or ‘Effective communication for contact lens instructions’ relate to the current guideline of ‘Communicate effectively with your patient’ from the GOC current standards of practice^36^ and ‘Disinfection of slit lamp’ would relate to the current guideline of ‘Ensure a safe environment for your patients’.^36^ Despite apparent clarity on how to deliver these elements of care in practice, other issues driven directly by, or in conjunction with, additional underlying factors are likely impacting practitioners.^37^ Understanding these relationships will be key to optimising the conditions for professionals to deliver safer outcomes. Interventions involving solely education and training for practitioners may only form part of a solution, and will need to be used as an element of a more comprehensive improvement effort to address wider systemic issues. Active monitoring, including use of the measurement and monitoring of safety framework^38^ in practice, will help to establish an environment for ongoing inquiry into the safety of care being delivered and reduce overreliance on compliance to pre‐existing guidelines.^39^

Incidents that were perceived to be avoidable that were charted under ‘other's control’ refer to other practitioners, members of staff in a practice, GPs and other eye care service providers. For these incidents, cross‐setting research may be required to address the aspect of the current system to improve the quality of care. Incidents such as ‘Glaucoma patient being sent a waiting list “validation letter” that got lost in the post so patient removed from list. Patient and optometrist not informed of removal’ indicates the interdependence of key functions in the care delivery for this patient group. Approaches to map non‐linear complex systems could help to understand the upstream and downstream implications of such incidents.

There were few incidents perceived to be unavoidable that were charted as ‘out of control’. For example, the likelihood of the incident ‘Theft/loss of medication’ could not be completely eliminated, but highlights an area for further investigations to consider increased safety measures and environmental factors as part of the wider system.

Several incidents suggested by participants aligned with the findings from studies that were based in secondary eye care. Suggested incidents involving patients being ‘lost to follow up’ and other incidents that would result in delays to patient care, incidents involving the ‘wrong eye’ and incidents involving the ‘wrong drug/drop used’ were also reported in several patient safety studies from secondary eye care.^7^, ^8^, ^10^, ^11^ This finding provides evidence that such incidents may be occurring across eye care services.

### Recommendations to improve practice, education and training

To address the need for a robust system to capture incident reports in the future, this research has informed the development of a new patient safety incident reporting system for use in primary eye care in Wales. The findings from this study have informed guidance for both the reporting system and a new training module for the profession on identifying and reporting patient safety incidents. Further classification of incidents that have occurred within eye care in Wales could maximise the understanding of patient safety in eye care. As shown in this study, identifying the incidents occurring most frequently in practice and those that professionals indicate could cause the most harm to patients highlights a helpful starting basis for further investigation to improve our understanding of how to design the structures, processes and environments to guide professionals to work safely in complex systems.

Diagnostic and administration‐related incidents have been identified as being the most frequent and severe incidents occurring in eye care in Wales. Whilst analysis of routinely reported patient safety incidents from optometrists is awaited, avoidable incidents within these categories should be considered for further exploration in the context of interventions to address the largest number of harmful incidents and improve the quality of care for patients.

### Strengths and limitations

This study is the first characterisation of perceived patient safety risks based on the experiences of eye care practitioners in Wales. The demographic data show that the optometrist and dispensing optician participants were mostly nationally representative of the population of eye care practitioners, notably when compared by sex, age/experience and the types of qualifications held. In addition, the range in length of practice of participants meant that a breadth of perspectives of incidents occurring in practice were likely captured. Data collection was undertaken anonymously to encourage genuine and truthful suggestions of incidents that had been observed in practice.

This study involved a limited sample of optometrists and dispensing opticians practicing in Wales, and therefore, the findings are not necessarily representative of the whole population of eye care service providers in the United Kingdom or internationally. The findings may have been limited by recall bias as they were solely reliant on the accuracy of the practitioners' memory and their ability to recall incidents they had seen in practice. This study is only representative of the nature of incidents from the perspectives of eye care practitioners as patients were not involved in the sample. A small amount of missing data were noted: 52 frequency votes were cast from 56 participants. There is also potential for volunteer bias within the sample, as the practitioners involved may already have an interest in patient safety or the quality of optometric care.

## CONCLUSION

Given the evidence of increased delays to patients with eye‐related problems, the findings of this work have reinforced the need for research into patient safety incidents occurring in eye care. A preliminary characterisation of patient safety incidents that have occurred in optometric practice was needed to inform the development of a new all‐Wales (UK) incident reporting system for use in primary care and training. Obtaining the views of incidents from practitioners within the eye care profession in Wales increases the understanding of current and previous issues affecting the quality of care being delivered. This work has identified several priority areas in eye care for further investigations, to improve the safety of patient care. The importance of understanding patient safety in eye care is paramount given the restructuring of eye care services and increasing clinical responsibility for optometrists.

## AUTHOR CONTRIBUTIONS

**Elinor MacFarlane:** Conceptualization (equal); data curation (lead); formal analysis (lead); methodology (equal); project administration (equal); validation (equal); writing – original draft (equal); writing – review and editing (equal). **Andrew Carson‐Stevens:** Conceptualization (equal); formal analysis (equal); funding acquisition (supporting); investigation (equal); methodology (equal); project administration (supporting); resources (supporting); supervision (supporting); writing – original draft (equal); writing – review and editing (equal). **Rachel North:** Conceptualization (supporting); data curation (supporting); formal analysis (supporting); investigation (supporting); methodology (supporting); project administration (supporting); supervision (supporting); writing – original draft (supporting); writing – review and editing (supporting). **Barbara Ryan:** Conceptualization (supporting); data curation (supporting); funding acquisition (supporting); project administration (supporting); resources (supporting); writing – review and editing (supporting). **Jennifer H. Acton:** Conceptualization (equal); data curation (supporting); formal analysis (equal); funding acquisition (lead); investigation (equal); methodology (equal); project administration (equal); resources (lead); supervision (lead); writing – original draft (equal); writing – review and editing (lead).

## CONFLICT OF INTEREST

The authors report no conflict of interest and have no proprietary interest in any of the materials mentioned in this article.

## Supporting information


Appendix S1
