# Supplementary material for: A mixed‐methods characterisation of patient safety incidents by primary eye care practitioners
Source: Ophthalmic Physiol Opt. 2022 Jul 31;42(6):1304–15. doi: 10.1111/opo.13030 (PMC9796726; doi:10.1111/opo.13030)
Supplement: Supplementary file 1 — Appendix S1 [file OPO-42-1304-s001.docx]

## Supplementary Information: A mixed-methods characterisation of patient safety incidents by primary eye care practitioners, MacFarlane et al.

Figure S.1: Age distribution of the optometrist workforce in Wales and the calculated age of workshop participants.

Figure S.2: The distribution of additional qualifications of participants and the optometrist workforce in Wales.

Figure S.3: The number of incidents within each theme, by incident category.

*Table S.1: Full table of quantitative data showing the number of incidents suggested and the numbers of votes received.*

| **Suggested Incidents** | **# of incidents suggested** | **# of ‘frequency’ votes** | **# of ‘severity’ votes** |
| --- | --- | --- | --- |
| **Medication** | | | |
| Wrong drug/drop used, wrong dose | 7 |  |  |
| Adverse reaction to medication | 4 |  |  |
| Incorrect prescription – no/poor checking | 4 | 3 |  |
| Not understanding patient’s true medication | 3 |  |  |
| Patient allergy/sensitivity not acted upon/asked | 3 |  |  |
| Use of Fluorescein on pregnant women | 2 |  |  |
| Angle closure induced | 2 |  |  |
| Peroxide used instead of contact lens solution | 2 |  |  |
| Wrong contact lens solution | 2 |  |  |
| Compliance with drops | 1 | 1 | 1 |
| Not communicating the consequences of an action | 1 |  |  |
| Mis-storage of medication | 1 |  |  |
| Misuse of an advised medication | 1 |  |  |
| GP reluctant to prescribe Glaucoma drops on repeat | 1 |  |  |
| Theft or loss of medication | 1 |  |  |
| Beta blockers and asthma | 1 |  |  |
| Wrong eye | 1 |  |  |
| Wrong regimen | 1 |  |  |
| **Total** | **38** | **4** | **1** |
| **Diagnosis** | | | |
| Not referring/timely referral/referral mishaps | 5 |  | 4 |
| Misdiagnosis – wrong techniques | 4 |  |  |
| Missed additional tests | 3 |  | 1 |
| Missed pathology | 2 |  | 22 |
| Not reviewing field tests | 2 | 1 |  |
| Failure to dilate | 2 |  | 1 |
| Unable to access eye when required | 1 |  | 7 |
| Not triaging unscreenable patients | 1 |  | 2 |
| Mis-stored images/scans/fields | 1 |  | 1 |
| Simple clinical mistakes | 1 |  |  |
| Failure to check corneas | 1 |  |  |
| Failure to attend to new symptoms | 1 |  |  |
| Patient reclassified as non-urgent after referral | 1 |  |  |
| Inappropriate treatment | 1 |  |  |
| Knowing correct referral route | 1 |  |  |
| Locum – pathways, hospitals | 1 |  |  |
| Not following up | 1 |  |  |
| Compliance with more advanced equipment | 1 |  |  |
| Wrong referral guideline | 1 |  |  |
| **Total** | **31** | **1** | **38** |
| **Communication** | | | |
| Referral/faxes not sent/received/wrong route/timescale | 8 | 4 | 4 |
| Miscommunication | 5 |  |  |
| Ensuring patient knows severity/risk of condition | 4 | 1 | 2 |
| Mental capacity issues | 3 |  | 1 |
| Follow up appointments/letters not received | 3 |  |  |
| Informed consent | 2 |  |  |
| Effective communication for contact lens instructions | 2 |  |  |
| Communicating treatment/regime | 2 |  |  |
| Letters sent to wrong department/speciality | 2 |  |  |
| Not advising to do dilation | 1 |  | 1 |
| Not discussing the risk associated with vision with the patient based on your findings e.g. found dry AMD, patient doesn’t know, therefore doesn’t know what symptoms to look out for & therefore fails to attend in a timely manner | 1 | 1 |  |
| Dispensing errors | 1 |  |  |
| Incomplete notes on referrals | 1 |  |  |
| Not showing optometrist triage quickly enough | 1 |  |  |
| Difficulty with triage on call | 1 |  |  |
| GP repeating prescription steroids | 1 |  |  |
| Phone in eye casualty | 1 |  |  |
| Not written communication -> discontinuation of medication | 1 |  |  |
| Reception – triage EHEW | 1 |  |  |
| Communication cascading from WL – HB – Practices - Clinicians | 1 |  |  |
| Lack of black box thinking | 1 |  |  |
| **Total** | **43** | **6** | **8** |
| **Equipment** | | | |
| Equipment not working/calibrated | 8 |  |  |
| Lack of equipment | 3 | 2 |  |
| Lack of training | 1 |  |  |
| Misuse of equipment | 1 |  |  |
| Fax | 1 |  |  |
| Foreign bodies | 1 |  |  |
| Disinfection | 1 |  |  |
| New techniques | 1 |  |  |
| Tonometer | 1 |  |  |
| **Total** | **18** | **2** | **0** |
| **Administration** | | | |
| Lost to follow up | 3 | 38 | 9 |
| Failures in triage | 3 |  |  |
| Endless waiting lists for IOP check | 1 | 1 |  |
| Knowing who to report the incident to | 1 |  |  |
| Incorrectly attaching notes to patient record | 1 |  |  |
| Incorrectly discharged from two services instead of one | 1 |  |  |
| Incorrect prescription given out and made externally | 1 |  |  |
| Loss of waiting list ‘validation letter’ and being removed | 1 |  |  |
| Failure to act on previously seen pathology | 1 |  |  |
| Inaccurate or inappropriate recall | 1 |  |  |
| Wrong GP surgery | 1 |  |  |
| GP changing drug requests | 1 |  |  |
| **Total** | **16** | **39** | **9** |
|  |  |  |  |
| **All incidents total** | **146** | **52** | **56** |

*Table S.2: Summary table of qualitative data.*

| **Themes and sub-theme** | **Medication** | **Diagnosis and clinical investigation** | **Communication** | **Equipment** | **Administration** |
| --- | --- | --- | --- | --- | --- |
| **Inappropriate clinical decision making** | 30 incidents – e.g. use of Fluorescein on pregnant women | 21 incidents – e.g. wrong techniques -> misdiagnosis | 14 incidents – e.g. wrong patient seen | 4 incidents – e.g. new techniques | 12 incidents – e.g. Failure to act on previously seen pathology |
| **Delayed or missed referral of patients to GPs or ophthalmologists** | 2 incidents – e.g. no access to medical records – practitioners not made properly aware of medication + potential conflicts | 10 incidents – e.g. patient reclassified as non-urgent after referral | 14 incidents – e.g. referral letter that hasn’t been sent | 1 incident – fax | 8 incidents – e.g. lost to follow up – within practices, from GPs |
| **Compromised communication with other practitioners or patients** | 18 incidents – e.g. misuse of an advised medication | 5 incidents – e.g. internal referral mishaps between practitioners (e.g. locum to resident & vice versa) | 28 incidents – e.g. effective communication for contact lens solution | 2 incidents – e.g. equipment not working – failure to make further arrangements to re-do | 11 incidents – e.g. GP changing drug requests |
| Patient adherence to guidance | 3 incidents – e.g. compliance with drops |  | 9 incidents – e.g. correct use of contact lens solution |  |  |
| **Delays in receiving eye care** | 8 incidents – e.g. wrong regimen | 23 incidents – e.g. inappropriate treatment | 18 incidents – e.g. follow up letter not received | 15 incidents – e.g. lack of equipment | 13 incidents – e.g. inaccurate/inappropriate recall |

*Table S.3: Summary table of incidents allocated by locus of control and harm severity.*

|  | **Medication** | **Diagnosis and clinical investigation** | **Communication** | **Equipment** | **Administration** |
| --- | --- | --- | --- | --- | --- |
| **Locus of control** | | | | | |
| Own control | 29 incidents – e.g. not adequately checking allergies or other medication that can lead to harm | 25 incidents – e.g. compliance with more advanced equipment DRP/OCT | 28 incidents – e.g. ensuring patient knows how serious their condition is | 15 incidents – e.g. equipment not calibrated | 7 incidents – e.g. knowing who to report the serious incident to |
| Other’s control | 15 incidents – e.g. GP prescribing Latanoprost instead of Lacrilube for dry eye | 9 incidents – e.g. Contact Lens Optician – not referring leading to reduced visual acuity | 17 incidents – e.g. GP repeating prescription steroids | 8 incidents – e.g. lack of appropriate equipment | 10 incidents – e.g. GP changing drug requests |
| Out of control | 7 incidents – e.g. side effects | 1 incident – unable to access eye casually when required | 11 incidents – e.g. faxes not getting there | 1 incident – failure of equipment/poor calibration | 3 incidents – e.g. lost to follow up – within practices, from GPs |
| **Harm severity** | | | | | |
| Impossible to classify | 3 incidents – e.g. Chloramphenicol, Zovirax prescribing | 1 incident – simple clinical mistakes | 4 incidents – e.g. lack of black box thinking | 2 incidents – e.g. tonometer | 1 incident – knowing who to report the serious incident to |
| No harm | 8 incidents – e.g. Tropicamide | 3 incidents – e.g. not doing OCT | 7 incidents – e.g. mental capacity issues | 14 incidents – e.g. calibration |  |
| Mild harm | 22 incidents – e.g. misuse of an advised medication | 14 incidents – e.g. Phone in eye casualty | 14 incidents – e.g. missing something | 4 incidents – e.g. foreign bodies – risks | 8 incidents – e.g. failure to triage properly |
| Moderate harm | 20 incidents – e.g. peroxide used instead of multipurpose contact lens solution | 20 incidents – e.g. not referring | 30 incidents – e.g. routine referrals – not received | 1 incident – disinfection – slit lamp | 10 incidents – e.g. delays to planned care, especially RI follow up |
| Severe harm | 4 incidents – e.g. angle closure induced | 12 incidents – e.g. unable to access eye casually when required | 8 incidents – e.g. referring urgent patients through the wrong route (e.g. AMD via GP) leaving delays | 1 incident – disinfection – slit lamp | 5 incidents – e.g. lost to follow up at Hospital Eye Service, glaucoma patient subsequently lost vision |
